# Anticoagulantes orais diretos no tratamento do tromboembolismo venoso em pacientes com câncer

**DOI:** 10.1590/1677-5449.080916

**Published:** 2016

**Authors:** Winston Bonetti Yoshida

**Affiliations:** 1 Universidade Estadual Paulista – UNESP, Faculdade de Medicina de Botucatu, Departamento de Cirurgia e Ortopedia, Botucatu, SP, Brasil.

Os novos anticoagulantes orais (NOACs) vêm sendo chamados mais recentemente de anticoagulantes orais diretos (DOACs), tendo em vista o foco de ação direto em sítios específicos da cascata de coagulação, como o fator Xa (rivaroxabana, apixabana e edoxabana) e o fator IIa (dabigatrana). Já a antivitamina K (AVK) age de forma indireta, assim como a heparina e seus derivados. Além disso, as pesquisas com os DOACs se iniciaram por volta do ano 2000 e, assim, estes já não são considerados tão novos^1^.

Essas drogas vêm sendo prescritas com frequência crescente para o tratamento do tromboembolismo venoso (TEV), tendo em vista fatores como via de administração oral em doses fixas, boa biodisponibilidade, meia-vida variando de 8 a 15 horas, sem necessidade de monitoramento^2^. A via de excreção renal varia conforme a droga, com 25% para apixabana, 33% para rivaroxabana, 50% para edoxabana e 80% para dabigatrana^2^. Em todas elas, o metabolismo é feito pela p-glicoproteína, sendo que para rivaroxabana e apixabana há envolvimento do citocromo P450-3A4. Em função disso, há uma série de interações medicamentosas com medicamentos que também utilizam essas vias metabólicas^3^. Como limitações, destacam-se a falta de antídotos e os preços em nosso meio. Nos casos de sangramento, a conduta recomendada é a suspensão das drogas (todas com meia-vida inferior à das AVKs), transfusões de sangue e concentrados de fatores da coagulação^4^. Para a dabigatrana, a diálise é uma opção devido à predominância de excreção renal; para a apixabana e a rivaroxabana, o uso de carvão ativado pode ser útil para a redução da absorção dessas drogas^2^^,^ 5.

Os DOACs foram licenciados para profilaxia do TEV em pacientes submetidos a cirurgias ortopédicas de grande porte^6^, prevenção de acidente vascular cerebral em pacientes com fibrilação atrial e tratamento do TEV^7^. Uma metanálise de estudos de fase III de tratamento do TEV com os DOACs mostrou eficácia e segurança similares ao tratamento convencional^8^.

O câncer é um fator de risco importante para o TEV, uma vez que está presente em todos os elementos da tríade de Virchow^9^. Pode interferir no fluxo sanguíneo através da possibilidade de aumento da viscosidade sanguínea e compressão tumoral. Com relação à lesão endotelial, pode invadir a parede vascular, aumentar o fator de von Willebrand e ativar a trombomodulina e interleucinas. Com relação à hipercoagulabilidade, pode promover aumento do fator tissular, do fibrinogênio e do inibidor da ativação da plasmina, além de diminuição da antitrombina e das proteínas C e S^9^.

Nos pacientes com TEV não provocado, cerca de 20 a 25% têm câncer associado. Por outro lado, nos pacientes com câncer, o risco de TEV aumenta de 6 a 7 vezes. Nos pacientes com metástases, o risco aumenta 3,2 vezes^10^^,^ 11.

As diretrizes para o tratamento de TEV associado ao câncer (CAT) recomendam que ele seja feito com heparina de baixo peso molecular (HBPM)^12^^,^ 13. Para os casos em que esse tratamento não é possível, as opções secundárias são AVK ou DOAC, sem preferência entre os dois^13^. Essa recomendação se deve à superioridade da HBPM em relação à AVK quanto à recorrência de TEV e pela similaridade de sangramento^12^^,^ 14 ^-^ 16. Além disso, ainda não foi feito nenhum estudo específico de CAT com os DOACs em comparação com as HBPMs. As informações disponíveis derivam de análises de subgrupos de tratamento de TEV dos estudos de Fase III com os DOACs, em que o comparador foi a AVK^17^.

Essas análises mostraram, em geral, similaridade de eficácia e segurança dos DOACs em relação à AVK nos subgrupos de pacientes com CAT e sem diferença entre os DOACs^18^. As limitações desses estudos incluem: ausência de comparação com as HBPMs (padrão-ouro), heterogeneidade entre as bases de dados dos estudos-fonte – sem estratificação dos tipos de TEV e de câncer – e a atividade ou não desta última doença no momento do tratamento. Portanto, os subgrupos avaliados podem não ser totalmente representativos do universo de pacientes com ambas as doenças (CAT)^18^. Finalmente, o tamanho da amostra dos subgrupos com câncer é inferior ao necessário para conclusões mais definitivas sobre eficácia e segurança^19^.

Assim, como conclusão, os estudos indicam que os DOACs parecem ser igualmente eficazes e seguros em comparação com a AVK em pacientes com CAT. No entanto, estudos clínicos dedicados a esses pacientes, incluindo como comparador as HBPMs, são necessários para obtenção de melhor nível de evidência nesses casos.
