# Supplementary material for: Anticoagulantes orais diretos no tratamento do tromboembolismo venoso em pacientes com câncer
Source: J Vasc Bras. 2016 Oct-Dec;15(4):263–4. [Article in Portuguese] doi: 10.1590/1677-5449.080916 (PMC5829722; doi:10.1590/1677-5449.080916)
Supplement: Translation

# Translation: Direct oral anticoagulants for treatment of venous thromboembolism in cancer patients

**Authors:** Winston Bonetti Yoshida

**Affiliations:** 1 Universidade Estadual Paulista – UNESP, Faculdade de Medicina de Botucatu, Departamento de Cirurgia e Ortopedia, Botucatu, SP, Brazil.

New oral anticoagulants (NOACs) have more recently come to be referred to as direct oral anticoagulants (DOACs) because of their direct focus of action on specific sites in the coagulation cascade, such as factor Xa (rivaroxaban, apixaban and edoxaban) and factor IIa (dabigatran). In contrast, antivitamin K (AVK) acts indirectly and so do heparin and it derivatives. Additionally, studies with DOACs began around the year 2000 and so they are no longer considered new.^1^

These drugs are being prescribed with increasing frequency to treat venous thromboembolism (VTE), because of factors such as oral administration in fixed doses, good bioavailability, half-lives varying from 8 to 15 hours, and no need for monitoring.^2^ The proportion of renal clearance varies from drug to drug, at 25% for apixaban, 33% for rivaroxaban, 50% for edoxaban, and 80% for dabigatran.^2^ In all of these, metabolism is via p-glycoprotein, while for rivaroxaban and apixaban the P450-3A4 cytochrome is also involved. This means that there is a series of drug interactions with other medications that also use these metabolic pathways.^3^ The most important limitations are the lack of antidotes and the prices charged in our setting (Brazil). The recommended approach for management of cases of bleeding is suspension of the drugs (all of which have a shorter half-life than AVKs), blood transfusions, and concentrated coagulation factors.^4^ Dialysis is another option in the case of dabigatran, since excretion is predominantly renal; while for apixaban and rivaroxaban, activated charcoal can be useful to reduce absorption.^2^^,^ 5

The DOACs have been licensed for VTE prophylaxis in patients undergoing major orthopedic surgery,^6^ for prevention of cerebral vascular accidents in patients with atrial fibrillation, and as a treatment for VTE.^7^ A meta-analysis of studies describing phase III trials of DOACs to treat VTE found that efficacy and safety are similar to conventional treatment.^8^

Cancer is an important risk factor for VTE, since it is present in all of the elements that make up Virchow’s triad.^9^ It can interfere in blood flow via increased blood viscosity and through compression by tumors. With regard to endothelial injury, it can invade the vessel wall, increase von Willebrand factor levels and activate thrombomodulin and interleukins. With regard to hypercoagulability, it can provoke increases in tissue factor, fibrinogen, and of plasmin activator inhibitor, and it can reduce levels of antithrombin and of C and S proteins.^9^

Around 20 to 25% of patients with unprovoked VTE have cancer. Seen from the opposite perspective, patients with cancer have a 6 to 7 times greater risk of VTE. In patients with metastases, the risk increases 3.2 times.^10^^,^ 11

The guidelines for treatment of cancer-associated thrombosis (CAT) recommend using low molecular weight heparin (LMWH).^12^^,^ 13 For cases in which this option is not possible, secondary choices are AVK or DOAC, with no preference for either.^13^ This recommendation is because LMWH is superior to AVK with relation to VTE recurrence and because they are similar with relation to bleeding.^12^^,^ 14 ^-^ 16 Furthermore, no studies specific to CAT have been conducted to compare DOACs with LMWHs. The information that is available has been derived from analyses of subsets of phase III trials of DOACs for VTE treatment, in which they were compared to AVK.^17^

In general, these analyses show that DOACs have similar efficacy and safety to AVK in subsets of patients with CAT and no differences between DOACs.^18^ Limitations of these studies include: lack of comparison with LMWHs (the gold standard), non-uniformity of source study databases – with no stratification of types of VTE or cancer – and differences in cancer activity or inactivity at the time of treatment. As a result, the subsets analyzed may not be entirely representative of the universe of patients with both diseases (CAT).^18^ Finally, the sample sizes of subsets with cancer were smaller than is needed for more definitive conclusions about efficacy and safety.^19^

As such, these studies concluded that DOACs appear to be equally effective and as safe as AVK in patients with CAT, but clinical studies dedicated to these patients and including comparison with LMWHs are needed to obtain results with the best evidence level for these cases.
